# A straightforward, environmentally beneficial synthesis of spiro[diindeno[1,2-b:2′,1′-*e*]pyridine-11,3′-indoline]-2′,10,12-triones mediated by a nano-ordered reusable catalyst

**DOI:** 10.1038/s41598-021-84209-6

**Published:** 2021-03-01

**Authors:** Mahsa Fathi, M. Reza Naimi-Jamal, Mohammad G. Dekamin, Leila Panahi, Oleg M. Demchuk

**Affiliations:** 1 Research Laboratory of Green Organic Synthesis and Polymers, Department of Chemistry, Iran University of Science and Technology, Tehran, 16846-13114 Islamic Republic of Iran; 2 Pharmaceutical Research Institute, 8 Rydygiera Street, 01-793 Warsaw, Poland

**Keywords:** Environmental chemistry, Materials science, Nanoscience and technology, Catalysis, Green chemistry, Inorganic chemistry, Materials chemistry, Organic chemistry, Chemical synthesis

## Abstract

A library of new spiro[diindeno[1,2-b:2′,1′-*e*]pyridine-11,3′-indoline]-2′,10,12-trione derivatives has been prepared in an efficient, one-pot pseudo four-component method mediated by a reusable heterogeneous nano-ordered mesoporous SO_3_H functionalized-silica (MCM-41-SO_3_H) catalyst. Excellent yields, short reaction times, as well as convenient non-chromatographic purification of the products and environmental benefits such as green and metal-free conditions constitute the main advantages of the developed synthetic methodology. The obtained fused indole-indenone dyes would be of interest to pharmaceutical and medicinal chemistry. Furthermore, due to their sensitivity to pH changes, they could be used as novel pH indicators.

## Introduction

Multicomponent reactions are extensively used as an efficient tool to construct complex molecular motifs of important pharmaceutics, plant protection compounds, functional materials, and building blocks of a variety of fine chemicals. Such methodology usually satisfies rigid requirements of green chemistry and is characterized by atom-economy, synthetic convergency, simple purification protocols, and decreased usage of expensive solvents and reagents^1–12^. In recent years, the multicomponent reaction strategy has been applied for the synthesis of spirooxindoles and other privileged isatine derivatives^13–20^.

**Figure 1:**
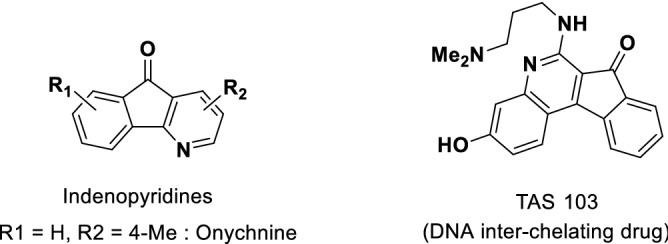
Examples of bioactive indenopyridines.

Many pharmaceutically important compounds possess a structural motif of merged indole and indenone cores^21–26^. The indenopyridine motif is found in many alkaloids and medicines, which exhibit anti-breast cancer^27^, cytotoxic^28–30^, calcium modulatory^31^, and other types of biological activities. At the same time, some such heterocycles are pH-indicators^32^, while others are used as building blocks in the synthesis of DNA inter-chelating drugs^33^ (Fig. 1).

A literature survey indicates a significantly small number of known methods for the synthesis of spiroindole core-based compounds. Several approaches to the synthesis of indenone-fused heterocycles from isatins, aromatic amines and 1,3-indanedione have been performed under various conditions, e.g. catalysis by *p*-toluenesulfonic acid^34,35^, sulfonated polyethylene glycol (PEG-OSO_3_H), *N*-methyl-2-pyrrolidonium dihydrogen phosphate ionic liquid^36^, oxalic acid dehydrate, a proline-based low transition temperature mixture^37^, and a zinc terephthalate metal–organic framework^38^. Despite some advantages, most of these methods have significant drawbacks, including the application of toxic, expensive solvents and catalysts, as well as complicated purification procedures and waste management protocols. Instead, heterogeneous recoverable catalysts used in the chemical industry and research labs make these processes much more environmentally friendly^39–43^.

In this study, we have focused on a heterogeneous acidic catalyst, which is easily prepared and has excellent activity and chemical stability. It can also be separated from the product after the reaction and be reused. Such parameters provide additional cost efficiency and environmental safety to the developing procedures^44–46^.

MCM-41 is a solid mesoporous nano-ordered silica with a large surface area and a regular structure. The diameter of the MCM-41 pores is distributed between 1.5 and 10 nm. It bears merely weak hydrogen bonding Si–OH sites and therefore at most only slightly acidic^47,48^. Its acidity could be improved, however, by substituting the Si atoms on its surface with Al^49^, B^50^, and Zn^51^, and or by functionalizing the MCM-41 surface with an alkyl sulfonic acid anchoring group^52,53^, succinamic acid^54^, or –SO_3_H^55–57^. Due to a large number of silanol groups, anchoring of inorganic –SO_3_H to the MCM-41 surface is very practical^58^. Such a readily accessible compound (MCM-41-SO_3_H) is non-toxic, recyclable, and reusable. Hence, MCM-41-SO_3_H is extensively applied in many chemical processes.

**Scheme 1:**
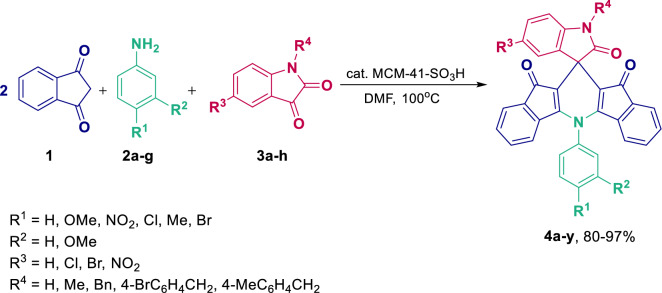
Synthesis of spiro[diindeno[1,2-b:2′,1′-*e*]pyridine-11,3′-indoline]-2′,10,12-triones.

As an extension of our continuous studies on the application of heterogeneous catalytic systems to the synthesis of different classes of pharmaceutically important compounds and to the development of green multicomponent reactions (MCRs)^57,59–64^, herein we report a straightforward approach leading to an effective, one-pot pseudo four-components synthesis of spiro[diindenopyridine-indoline]triones. The reaction between 1,3-indandione (**1**), aromatic amines (**2a-g**), and isatins (**3a-h**) in DMF is catalyzed by MCM-41-SO_3_H affording spiro[diindenopyridine-indoline]triones with good to excellent yields (Scheme 1).

## Results and discussion

### Characterization of the MCM-41-SO_3_H

**Figure 2:**
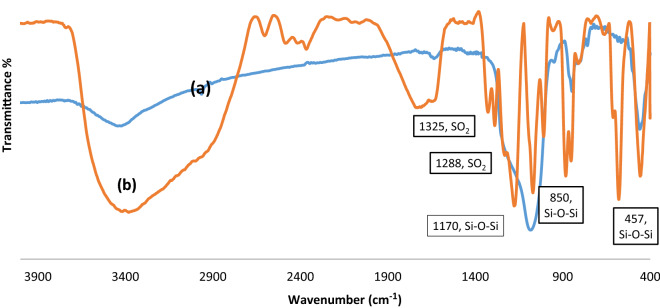
FT-IR spectra of nano-ordered MCM-41 (**a**), and MCM-41-SO_3_H (**b**).

The MCM-41-SO_3_H was prepared according to our previous reports^57,59,60^ and characterized by Fourier transform infrared spectroscopy (FT-IR), scanning electron microscopy (SEM), and Brunauer–Emmett–Teller analysis (BET). The FTIR spectrum of the catalyst has been shown in Fig. 2. The bands at 1325 and 1288 cm^−1^ correspond to the asymmetric and symmetric stretching vibrations of the SO_3_H group. A wide band in the area of 3400–3200 cm^−1^ is related to the O–H stretching vibration of the hydroxyl groups. Moreover, stretching vibrations of Si–O–Si are indicated by sharp bands at 1170 and 850 cm^−1^^65,66^.

**Figure 3:**
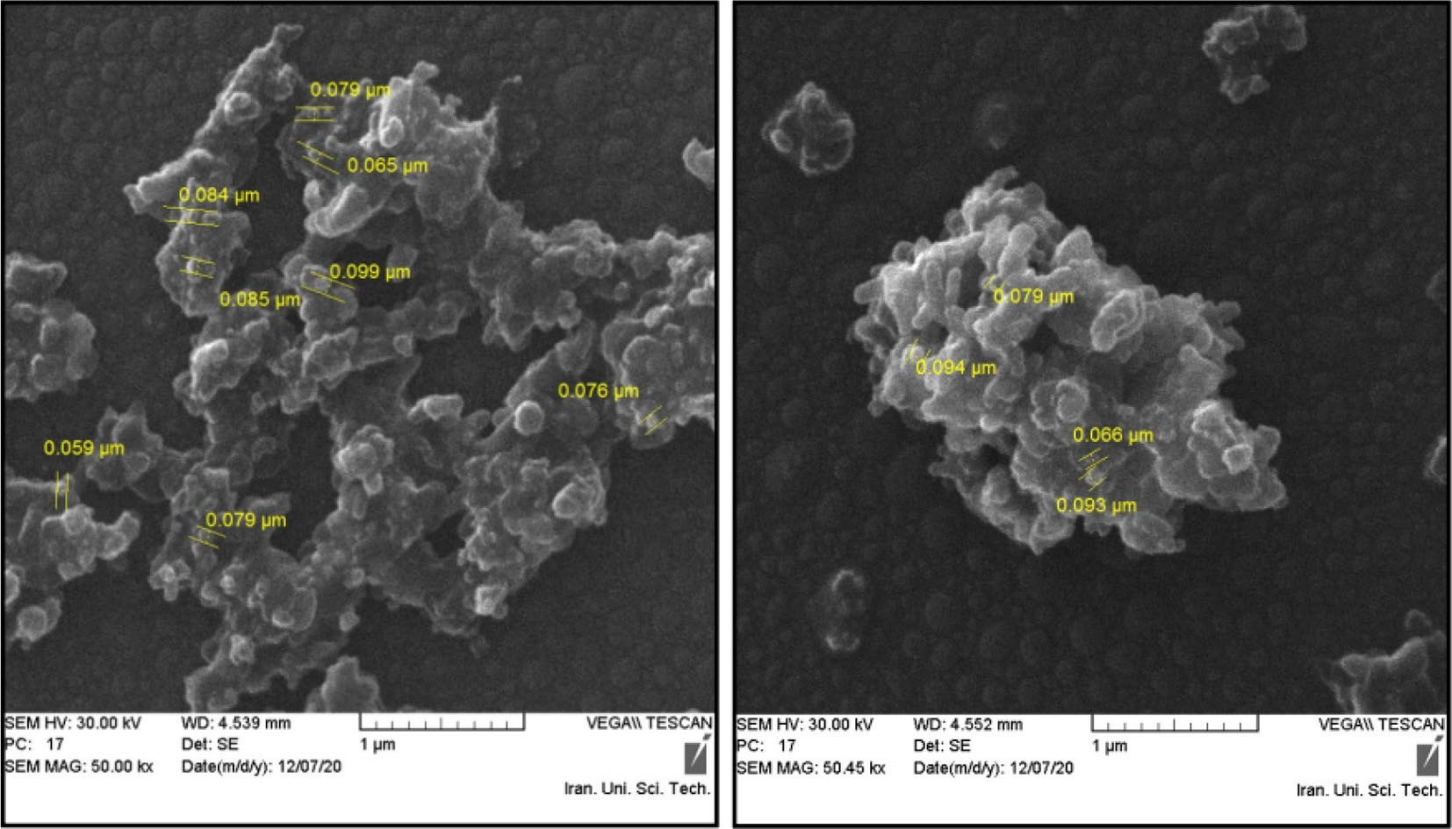
The SEM images of the MCM-41-SO_3_H: the fresh catalyst (left), and the recovered one (right).

**Figure 4:**
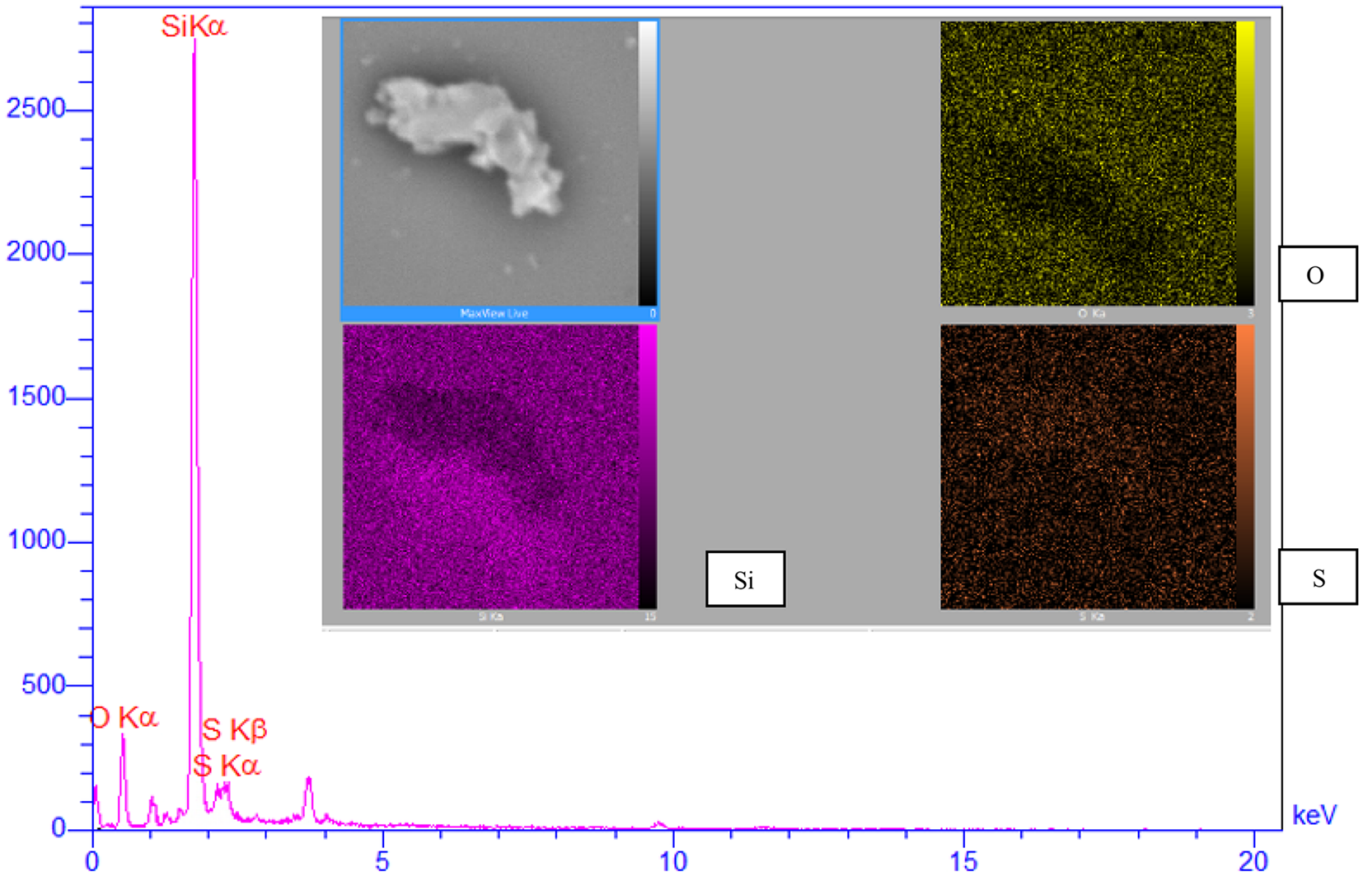
The EDX analysis of the fresh MCM-41-SO_3_H.

The SEM images of the fresh and recovered MCM-41-SO_3_H have been shown in Fig. 3 and confirm the nanoscale size of the synthesized particles. The size of most particles was in the range of 50–90 nm. As can be seen, the particles are aggregated, due to the strong hydrogen bonding between the acidic moieties. The EDX analysis of the fresh catalyst proved the presence of O, Si, and S atoms in the MCM-41-SO_3_H structure with a uniform distribution of the sulfonic acid groups (Fig. 4).

**Figure 5:**
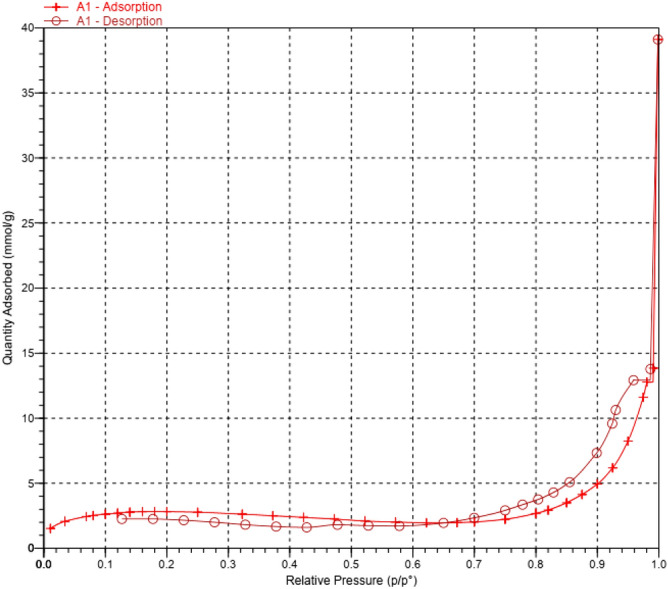
The N_2_ adsorption–desorption isotherms of the MCM-41-SO_3_H.

According to the obtained results from the N_2_ adsorption–desorption diagram (Fig. 5), the BET and the Langmuir surface area of the MCM-41-SO_3_H were 223 and 303 m^2^ g^−1^, respectively. The BET adsorption average pore width (4 V/A) was measured to be 7.2 nm. The catalyst surface area and porosity properties are in good agreement with a typical mesoporous material.

### Synthesis of spiro[diindeno[1,2-b:2′,1′-e]pyridine-11,3′-indoline]-2′,10,12-triones: an optimization of the reaction conditions

As it was mentioned above, our study aimed to discover an environmentally benign protocol for the synthesis of diversified spiro[diindeno[1,2-b:2′,1′-e]pyridine-11,3′-indoline]-2′,10,12-triones, which would satisfy the requirements of green chemistry^67,68^. Desired products were obtained in one-pot multicomponent reactions between 1,3-indanedione (**1**), anilines (**2**), and isatins (**3**). To find optimal conditions, several variables affecting the reaction yield were assessed.

### Assessment of the effect of the catalyst used

The catalytic efficiency of various members of the MCM-41 family (Al-MCM-41, Fe-MCM-41, MCM-41-NH_2_, and MCM-41-SO_3_H), as well as several other solid acid catalysts such as cellulose-SO_3_H, pectin, carboxymethyl cellulose, and hydroxyapatite were compared. The results presented in Table 1 indicate that a higher yield of the benchmark reaction between **1**, **2a**, and **3a**, which furnished 5-(phenyl)-5H-spiro[diindeno[1,2-b:2′,1′-e]pyridine-11,3′-indoline]-2′,10,12-trione (**4a**) was observed, when MCM-41-SO_3_H was used. The effectiveness of this catalyst could be rationalized taking the high Brönsted acidity of the catalyst used and its appropriate pore size into consideration. The loading of MCM-41-SO_3_H was also optimized. The yields of 64% and 89% **4a** were obtained when 5 and 10 mg of MCM-41-SO_3_H catalyst were used in a 1 mmol scale of the reaction at 100 °C for 2 h (Table 1, entries 11 and 12); whereas a 94% yield was achieved after the first 20 min of the reaction in the presence of 20 mg of the catalyst (Table 1, entry 6).

**Table 1:** The evaluation of activity of different catalysts in the model reaction.


Entry	Catalyst (mg)	Time, h	Isolated yield, %
1	–	24	< 30
2	Al-MCM-41 (20)	24	37
3	Fe-MCM-41 (20)	24	52
4	MCM-41-NH_2_ (20)	24	< 30
5	MCM-41 (20)	24	57
6	MCM-41-SO_3_H (20)	1/3	94
7	Cellulose-SO_3_H (20)	1	78
8	Pectin (20)	24	47
9	Carboxymethyl cellulose (20)	24	< 30
10	Hydroxyapatite (20)	24	< 30
11	MCM-41-SO_3_H (5)	2	64
12	MCM-41-SO_3_H (10)	2	89

Reaction conditions: isatin (1 mmol), 1,3-indanedione (2 mmol), aniline (1 mmol) and DMF (1 mL) at 100 °C.

### Assessment of the effect of the solvent and temperature

The effect of various polar and non-polar, protic and aprotic solvents (EtOAc, *n*-hexane, EtOH, CH_3_CN, DMF, and DMSO) on the yield of the model reaction was also evaluated (Table 2, entries 1 and 4–8).

**Table 2:** The evaluation of different solvents and reaction temperatures in the model reaction.


Entry	Solvent	Catalyst (mg)	Time, h	Temperature, °C	Isolated yield, %
1	DMF	MCM-41-SO_3_H (20)	1/3	100	94
2	DMF	MCM-41-SO_3_H (20)	2	60	74
3	DMF	MCM-41-SO_3_H (20)	1/2	80	90
4	n-hexane	MCM-41-SO_3_H (20)	12	Reflux	< 30
5	EtOAc	MCM-41-SO_3_H (20)	12	Reflux	< 30
6	CH_3_CN	MCM-41-SO_3_H (20)	4	Reflux	84
7	DMSO	MCM-41-SO_3_H (20)	1/3	100	94
8	EtOH	MCM-41-SO_3_H (20)	12	Reflux	65
9	Ball milling (solvent-Free)	MCM-41-SO_3_H (20)	10	RT	42

Reaction conditions: isatin (1 mmol), 1,3-indanedione (2 mmol), aniline (1 mmol) and solvent (1 mL).

The polar aprotic solvent DMF was found to be a solvent of choice in this reaction (Table 2, entry 1). The solvent-free reaction ran in identical conditions -but without any solvent- with a poor yield, event after a long reaction time (Table 2, entry 9). On the other hand, the evaluation of the temperature influence on the yield of **4a** indicated that higher temperature resulted in an improved yield in a shorter reaction time (Table 2, entries 1–3).

In comparison with other catalysts used in the similar reaction reported previously (Table 3), the heterogeneous MCM-41-SO_3_H was beneficial, offering higher sustainability and better efficiency in the synthesis of **4**a. In addition, the atom economy of the protocol proposed herein and the waste exclusion proved its greenness as well.

**Table 3:** The efficiency of MCM-41-SO_3_H as compared to other reported catalysts in the model reaction.


Entry	Reaction conditions	Time, min	Yield, %	References
1	PTSA/62 mg/grinding/	3–4	85	^34^
2	PTSA/30 mol%/CH_3_CN/reflux	60	82	^35^
3	PEG-OSO_3_H/30 mol%/80 °C	10	94	^36^
4	[NMP]H_2_PO_4_/30 mol%/80 °C	15	92	^36^
5	LTTM/excess/80 °C	20	94	^37^
6	Zn (BDC) MOF/5 g/80 °C	25	96	^38^
7	MCM-41-SO_3_H/20 mg/100 °C	20	95	Present work

### Assessment of the substrate scope

In general, high to excellent yields of spiro[diindeno[1,2-b:2′,1′-e]pyridine-11,3′-indoline]-2′,10,12-triones **4a-y** with a broad range of substituents were achieved in a short reaction time (Table 4).

**Table 4:** Synthesis of spiro[diindeno[1,2-*b*:2′,1′-*e*]pyridine-11,3′-indoline]-2′,10,12-triones.


Entry	R^1^	R^2^	R^3^	R^4^	Product	Time, min	Yield, %	Melting point, °C
1	H	H	H	H	**4a**	20	95	> 300
2	OMe	H	H	H	**4b**	15	97	> 300
3	NO_2_	H	H	H	**4c**	25	90	> 300
4	Cl	H	H	H	**4d**	20	92	> 300
5	Me	H	H	H	**4e**	18	95	> 300
6	Br	H	H	H	**4f**	20	92	> 300
7	H	H	Cl	H	**4g**	20	92	> 300
8	OMe	H	Cl	H	**4h**	15	94	> 300
9	Me	H	Cl	H	**4i**	18	93	> 300
10	Cl	H	Cl	H	**4j**	20	91	> 300
11	H	H	Br	H	**4k**	25	90	> 300
12	OMe	H	Br	H	**4l**	18	94	> 300
13	Me	H	Br	H	**4m**	20	93	> 300
14	Cl	H	Br	H	**4n**	22	92	> 300
15	H	H	NO_2_	H	**4o**	30	89	> 300
16	OMe	H	NO_2_	H	**4p**	20	90	> 300
17	Me	H	NO_2_	H	**4q**	20	90	> 300
18	Cl	H	NO_2_	H	**4r**	25	88	> 300
19	H	H	H	Me	**4s**	22	89	> 300
20	OMe	H	H	Me	**4t**	20	92	> 300
21	Me	H	H	Me	**4u**	20	90	> 300
22	Cl	H	H	4-Tol	**4v**	25	89	> 300
23	OMe	H	H	Bn	**4w**	30	85	> 300
24	OMe	H	H	4-BrC_6_H_4_CH_2_	**4x**	35	80	> 300
25	H	OMe	H	H	**4y**	15	94	> 300

Reaction conditions: isatin (**1**, 1 mmol), 1,3-indanedione (**2**, 2 mmol) and aniline (**3**, 1 mmol), MCM-41-SO_3_H (20 mg), DMF (1 mL), 100 °C.

In comparison with the EWG-substituted substrates (Table 4, entries 3, 15–18), higher yields and shorter reaction times were observed for EDG-substituted isatins and aromatic amines (Table 4, entries 2, 5, 8, 12, 16).

### The study on catalyst stability and reusability

**Figure 6:**
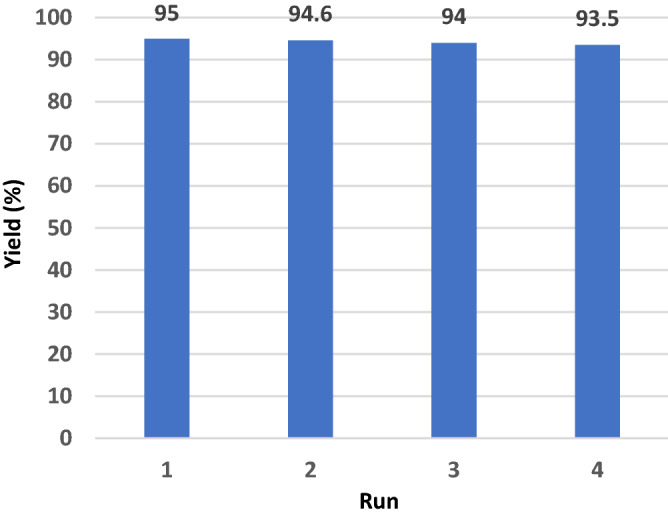
Reusability of the MCM-41-SO_3_H catalyst in four consecutive runs.

The possibility of recovering and reusing the catalyst was assessed in four consecutive runs for the benchmark reaction leading to **4a**. After each run, the catalyst was filtered off and washed with *n*-hexane and acetone. Next, it was dried at 60 °C for 0.5 h. The recycled catalyst was then subjected to the next run of the model reaction. A significant maintaining of the catalytic activity of MCM-41-SO_3_H in each run of the reaction was observed (Fig. 6).

**Figure 7:**
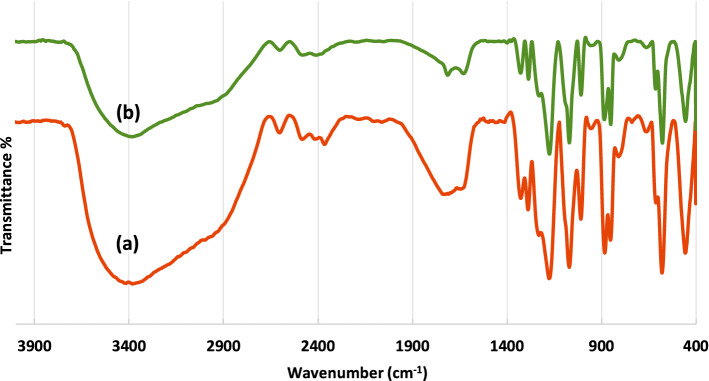
The FT-IR spectra of the fresh MCM-41-SO_3_H (**a**), and the recovered one (**b**).

The FT-IR spectra of the fresh and the recovered MCM-41-SO_3_H catalyst after the fourth run indicated that its structure remained unchanged (Fig. 7).

### Possible mechanism

**Scheme 2:**
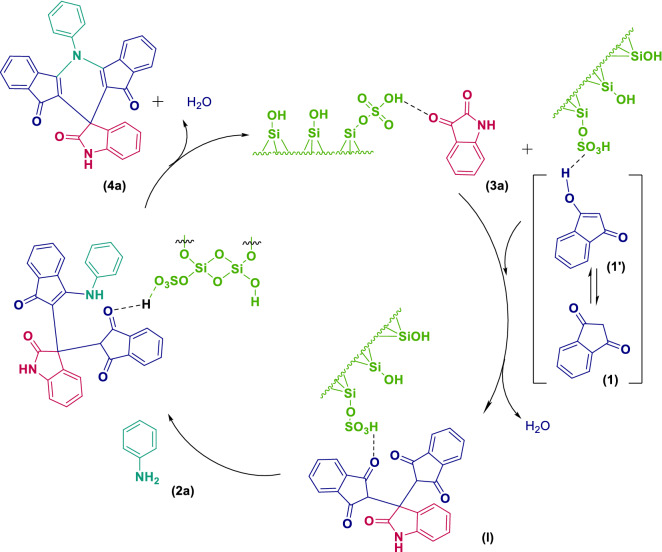
Proposed catalytic role of the MCM-41-SO_3_H in the multicomponent condensation leading to the spirodiindenopyridine indoline **4a**.

A plausible mechanism of the reaction leading to spiro[diindenopyridine-indoline]triones is outlined in Scheme 2.

According to the proposed mechanism, acidic SO_3_H groups located on the surface of MCM-41-SO_3_H activate the carbonyl group of isatin (**3a**) to facilitate initial nucleophilic addition of the enolic form of 1,3-indanedione (**1**) affording the intermediate **I**. The addition of **2a** to the intermediate **I**, followed by a cyclization reaction, furnishes the product **4a**.

Study of the spectral properties of the obtained products **4a**–**4y**.

The UV–Vis spectra of **4a–4y** were obtained in methanol and reported in Table 5. They showed a maximum absorption wavelength (λ_max_) in the range of 422–435 nm and a molar extinction coefficient (ɛ) of (1.08–2.99) × 10^5^ L mol^−1^ cm^−1^.

**Table 5:** Spectral properties of the obtained products **4a**–**4y**.

Compound	λ_max_, nm	ε, 10^5^ L mol^−1^ cm^−1^	Entry	λ_max_, nm	ε, 10^5^ L mol^−1^ cm^−1^
**4a**	435	1.76	**4n**	424	1.79
**4b**	437	1.08	**4o**	429	2.76
**4c**	425	1.08	**4p**	432	2.99
**4d**	430	1.74	**4q**	424	2.08
**4e**	432	1.31	**4r**	435	2.65
**4f**	431	1.87	**4s**	432	2.11
**4g**	428	1.42	**4t**	426	2.55
**4h**	432	1.52	**4u**	428	2.84
**4i**	429	1.15	**4v**	438	2.93
**4j**	424	1.76	**4w**	435	2.84
**4k**	425	1.91	**4x**	426	2.69
**4l**	427	2.86	**4y**	434	2.54
**4m**	422	2.05			

Recorded in methanol solution.

**Figure 8:**
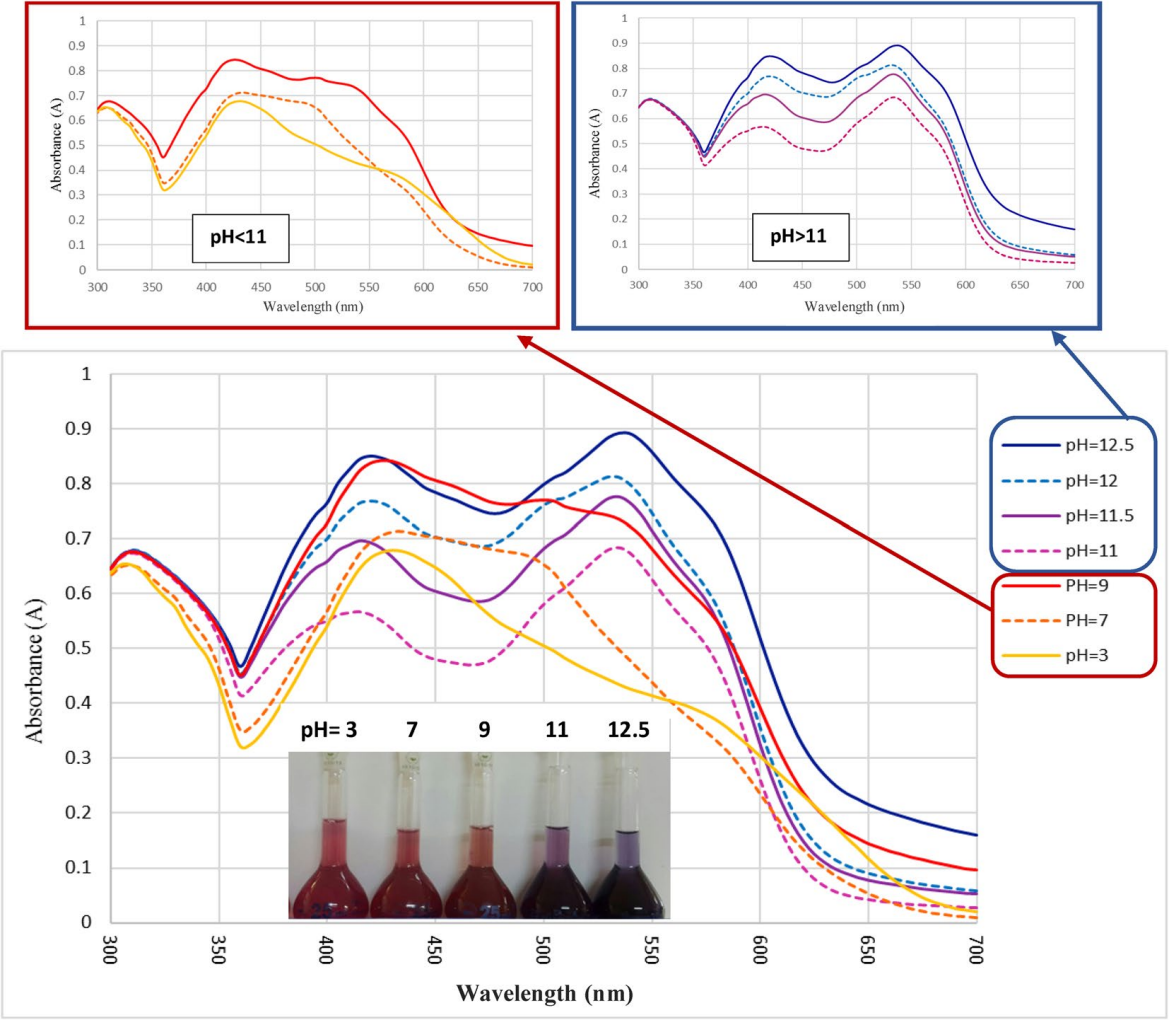
Spectral changes of the 5-(phenyl)-5H-spiro[diindeno[1,2-*b*:2′,1′-*e*]pyridine-11,3′-indoline]-2′,10,12-trione (**4a**) (Metanol, 100 ppm) at different pH values.

The Spiro[diindenopyridine indoline]triones with a hydrogen atom at indoline nitrogen may undergo reversible deprotonation and can be used as pH chemo-sensors. The product **4a** was examined as a pH indicator and showed a visible color change at pH ca. 11, from red (in the acidic media) to deep blue (in the basic conditions) (Fig. 8). However, the solution of *N*-substituted isatins (e.g. **4w)** showed no remarkable color change in an alkali solution.

**Scheme 3:**
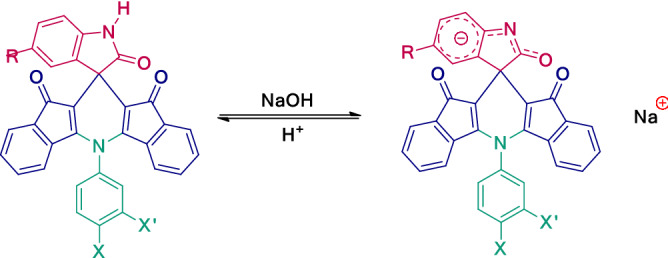
The pH effect on the Π-system of spirodiindenopyridine indolines derivatives.

The UV–Vis absorption of the compound **4a** was measured in the pH range from 3 to 12.5. As shown in Fig. 8, beginning from *ca*. pH 9, a second absorption peak around 530 nm appears. The spectral data is given in Table 6. Whereas the N–H isatins solution displayed similar behavior, *N*-substituted isatins showed no color change in a wide range of pH. It seems that the color change in dye **4a** is due to the deprotonation of the NH group in the indoline unit (Scheme 3).

**Table 6:** Influence of pH on the UV–Vis absorption of the dye **4a**.

pH	λ_max_1 (nm)	λ_max_2 (nm)
3	430	–
7	435	–
9	425	–
11	415	530
11.5	415	530
12	420	530
12.5	420	535

## Conclusions

To summarize, we have herein reported a straightforward atom-economical method of synthesis of spiro[diindenopyridine indoline]triones mediated by a safe heterogeneous recyclable catalyst MCM-41-SO_3_H, which could be used for at least 4 runs without any significant loss of its activity. Among other advantages of the current protocol, we could emphasize excellent yields, short reaction times, high atom economy as well as simple isolation and purification procedures for both the catalyst and products. The presented approach leading to spiro[diindenopyridine-indoline]triones can be of interest to medicinal and pharmaceutical chemistry. Furthermore, some products exhibit a pH indicator activity proven by a visible color change in the basic pH ranges (Supplementary Information S1).

## Supplementary Information

Supplementary Information
