# Supplementary material for: A straightforward, environmentally beneficial synthesis of spiro[diindeno[1,2-b:2′,1′-e]pyridine-11,3′-indoline]-2′,10,12-triones mediated by a nano-ordered reusable catalyst
Source: Sci Rep. 2021 Mar 1;11:4820. doi: 10.1038/s41598-021-84209-6 (PMC7921678; doi:10.1038/s41598-021-84209-6)
Supplement: Supplementary file 1 — Supplementary Information [file 41598_2021_84209_MOESM1_ESM.docx]

***Electronic Supporting Information***

**A straightforward, environmentally beneficial synthesis of spiro[diindeno[1,2-b:2′,1′-*e*]pyridine-11,3′-indoline]-2′,10,12-triones mediated by a nano-ordered reusable catalyst**

**Mahsa Fathi^a^, M. Reza Naimi-Jamal*^a^, Mohammad G. Dekamin^a^,**

**Leila Panahi^a^, Oleg M. Demchuk^b^**

*^a^ Research Laboratory of Green Organic Synthesis & Polymers, Department of Chemistry, Iran University of Science and Technology, Tehran 16846-13114, I. R. Iran.*

*^b^ Pharmaceutical Research Institute, 8 Rydygiera Street, 01-793 Warsaw, Poland*

**Email:* [*naimi@iust.ac.ir*](mailto:naimi@iust.ac.ir)*; Fax: +98-21-77491204*

**Experimental Section**

**Materials and instrumentation**

All commercially available compounds were purchased from Merck KGaA and used without further purification. The progress of the reaction was monitored by TLC using Merck 0.2 mm silica gel 60 F-254 precoated Al-plates eluted with EtOAc / *n*-hexane solvent mixtures. Melting points were measured on the Electrothermal 9100 apparatus and are uncorrected. The ^1^H NMR and ^13^C NMR spectra were recorded on a BRUKER DRX-300 AVANCE spectrometer in DMSO-*d_6_*, at ambient temperature. The coupling constants (*J*) are in Hertz and the chemical shift values were recorded on the δ scale. The FT-IR spectra were recorded for KBr pellets on a Shimadzu FTIR-8400S spectrometer. High-resolution mass measurements (HRMS) were performed on a Shimadzu ITTOF LCMS spectrometer, the ESI was applied. N_2_ adsorption-desorption isotherms were measured on a Micromeritics ASAP 2020 at 77 K. BET sorptometer for surface area and pore size analysis was carried out using PMI's BET Sorptometer.

**General procedure for the preparation of MCM-41**

MCM-41 was prepared according to a known procedure described in our previous publication ^57^. To the solution of 2.70 g of diethylamine in 42 mL of water, stirred in a 200 mL beaker at room temperature, 1.47 g of cetyltributylammonium bromide (CTAB) was added. In approximately 30th min of stirring, after the desolvation had been completed, 2.10 g of tetraethyl orthosilicate (TEOS) was added drop-wise. After that, the pH of the reaction mixture was adjusted to 8.5 by slowly adding 1 M HCl. The reaction mixture was stirred for an additional 2 h and the solid product was filtered off. It was washed with water several times, dried at 45 °C for 12 h, and eventually calcined at 550 °C for 5 h ^57^.

**General procedure for preparation of MCM-41-SO_3_H**

A 100 mL suction flask, equipped with a gas outlet tube (for liberating HCl gas) and a constant pressure dropping funnel containing a solution of 2 mL of chlorosulfonic acid in 15 mL of CH_2_Cl_2_, was loaded with a suspension of 1.0 g of MCM-41 in 5 mL of CH_2_Cl_2_. Then, the solution of ClSO_3_H was slowly dropped in over a period of 30 min at room temperature (Caution: HCl gas was liberated!). When the addition of ClSO_3_H was completed, the mixture was stirred for an additional 30 min and the solvent was evaporated off under reduced pressure to obtain the light grey solid MCM-41-SO_3_H ^57^.

**Typical procedure for MCM-41-SO_3_H-mediated synthesis of spiro[diindeno[1,2-b:2′,1′-*e*]pyridine-11,3′-indoline]-2′,10,12-triones**

A 5 mL round-bottomed flask was loaded with 1,3-indanedione (**1**) (0.292 g, 2 mmol), aniline (**2a**) (93 mg, 1 mmol), isatin (**3a**) (147 mg, 1 mmol), DMF (1 mL), and MCM-41-SO_3_H (20 mg). The obtained mixture was stirred and heated in an oil bath at a fixed 100 °C temperature for the time mentioned in Table 2. (monitored by TLC, eluent: *n*-hexane: EtOAc = 1:1) When the reaction was completed, it was cooled down to an ambient temperature. Then, MCM-41-SO_3_H was separated by filtration and washed with CH_2_Cl_2_. Combined organic phases were washed with 5 mL of water, and dried over anhydrous MgSO_4_. After the evaporation of the solvent, the obtained solid was crystallized from the mixture EtOH/CH_3_CN to afford the pure product.

**Characterization spectral data for selected and new pigments (4b, 4v, 4w, 4x and 4y):**

**1)** 5-(4-Methoxyphenyl)-5*H*-spiro[diindeno[1,2-b:2′,1′-*e*]pyridine-11,3′-indoline]-2′,10,12-trione **(4b)**:

Yield: 97 %; Red powder. Mp > 300 °C; ^1^H NMR (300 MHz, DMSO-*d_6_*): δ (ppm) 3.97 (s, 3H, OCH_3_), 5.60 (d, *J =* 9.0 Hz, 2H, Ar-H), 6.84 (d, *J =* 9.0 Hz, 2H, Ar-H), 6.99 – 7.26 (m, 10H, Ar-H), 7.64 – 7.68 (m, 1H, Ar-H), 7.83 (d, 1H, Ar-H), 10.58 (s, 1H, NH); FT IR (KBr pellet, cm^−1^): 3371, 3058, 1693, 1618, 1386, 1251, 1174 ^38^.

**
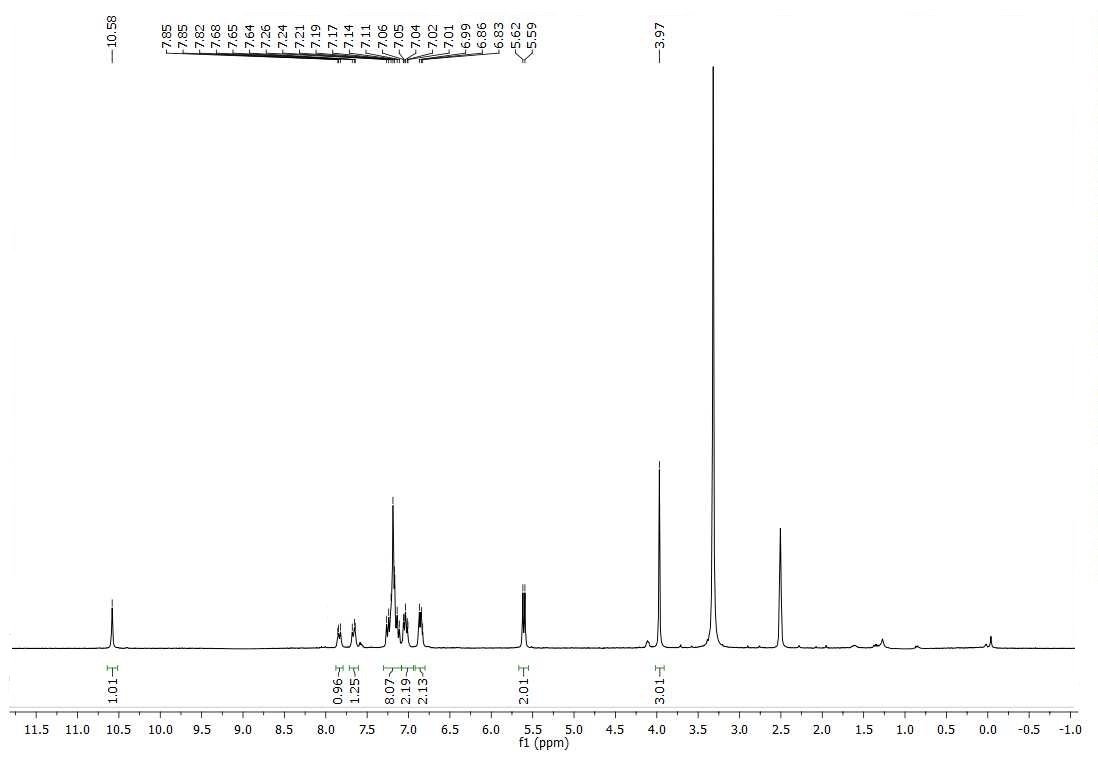
**

**Fig. 1.** ^1^H NMR spectrum of compound **4b** in DMSO-*d_6_.*

**Fig. 2.** FTIR spectrum of compound **4b** (KBr pellet).

**2)** 5-(4-Chlorophenyl)-1′-(*p*-tolyl)-5*H*-spiro[diindeno[1,2-*b*:2′,1′-*e*]pyridine-11,3′-indoline]-2′,10,12-trione **(4v)**:

Yield: 89 %; Red powder. Mp > 300 °C; ^1^H NMR (300 MHz, DMSO-*d_6_*): δ (ppm) 2.50 (s, 3H, CH_3_), 5.61 (d, *J =* 6.0 Hz, 2H, Ar-H), 6.66–6.69 (m, 1H, Ar-H), 6.90 – 6.95 (m, 3H, Ar-H), 7.08 – 7.45 (m, 8H, Ar-H), 7.60 – 7.65 (m, 2H, Ar-H), 7.66 – 7.68 (m, 1H, Ar-H), 7.77 – 7.80 (m, 3H, Ar-H); ^13^C NMR (75 MHz, DMSO-*d_6_*): δ (ppm) 38.78, 39.13, 39.45, 39.73, 40.57, 40.85, 45.66, 48.13, 111.87, 116.11, 120.08, 122.17, 123.24, 123.60, 124.00, 124.38, 125.42, 127.52, 128.95, 130.01, 131.12, 132.86, 133.23, 133.97, 134.29, 135.33, 136.09, 138.20, 139.98, 148.18, 156.74, 174.54, 190.23; FT IR (KBr pellet, cm^−1^): 3060, 1693, 1616, 1478, 1386, 1236, 1174; HRMS (ESI): *m/z* [M + H]^+^ calcd. for C_39_H_24_ClN_2_O_4_: 602.1190, found: 602.1205.

**
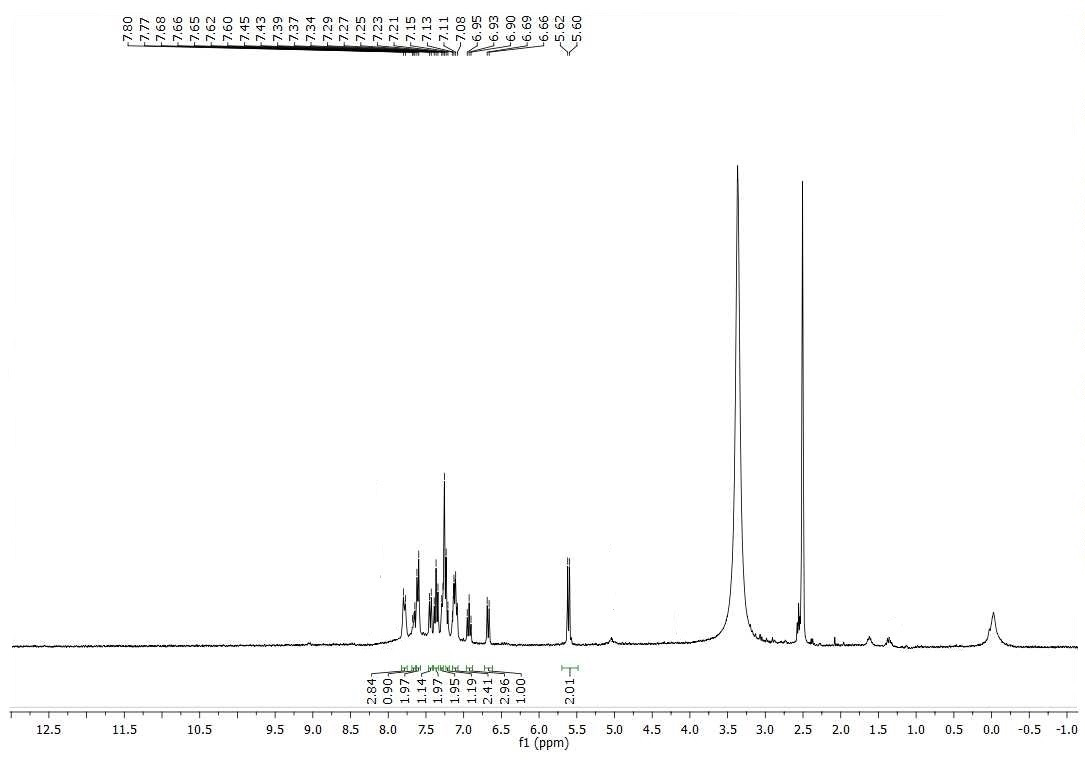
**

**Fig. 3.** ^1^H NMR spectrum of compound **4v** in DMSO-*d_6_.*

**
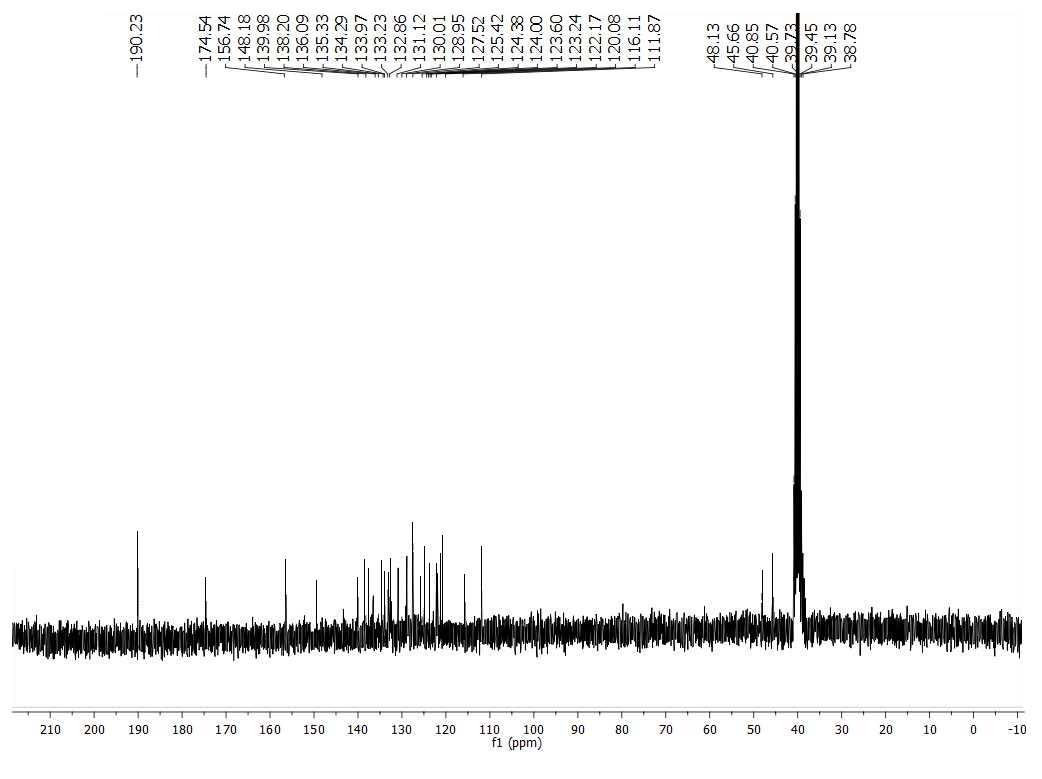
**

**Fig. 4.** ^13^C NMR spectrum of compound **4v** in DMSO-*d_6_.*

**Fig. 5.** FTIR spectrum of compound **4v** (KBr pellet).

**3)** 1′-Benzyl-5-(4-methoxyphenyl)-5*H*-spiro[diindeno[1,2-*b*:2′,1′-*e*]pyridine-11,3′-indoline]-2′,10,12-trione **(4w)**:

Yield: 85 %; Red powder; Mp > 300 °C; ^1^H NMR (300 MHz, DMSO-*d_6_*): δ (ppm) 3.97 (s, 3H, OCH_3_), 5.02 (s, 2H, CH_2_), 5.62 (d, *J =* 9.0 Hz, 2H, Ar-H), 6.62 (d, *J =* 9.0 Hz, 2H, Ar-H), 6.90 – 6.93 (m, 1H, Ar-H), 6.99 – 7.02 (m, 1H, Ar-H), 6.90 – 7.32 (m, 9H, Ar-H), 7.34 – 7.37 (m, 2H, Ar-H), 7.56 – 7.59 (m, 2H, Ar-H), 7.65 (m, 1H, Ar-H), 7.68 (m, 1H, Ar-H); ^13^C NMR (75 MHz, DMSO-*d_6_*): δ (ppm) 56.33, 57.95, 59.50, 102.39, 118.16, 125.75, 126.59, 127.24, 127.92, 128.34, 129.22, 129.67, 138.39, 138.87, 139.60, 140.08, 140.80, 146.32, 151.94, 152.29, 169.84, 193.22; FT IR (KBr pellet, cm^−1^): 3058, 1695, 1616, 1508, 1384, 1251, 1174; HRMS (ESI): *m/z* [M + Na]^+^ calcd. for C_40_H_26_N_2_O_4_: 621.1785, found: 621.1801.

**
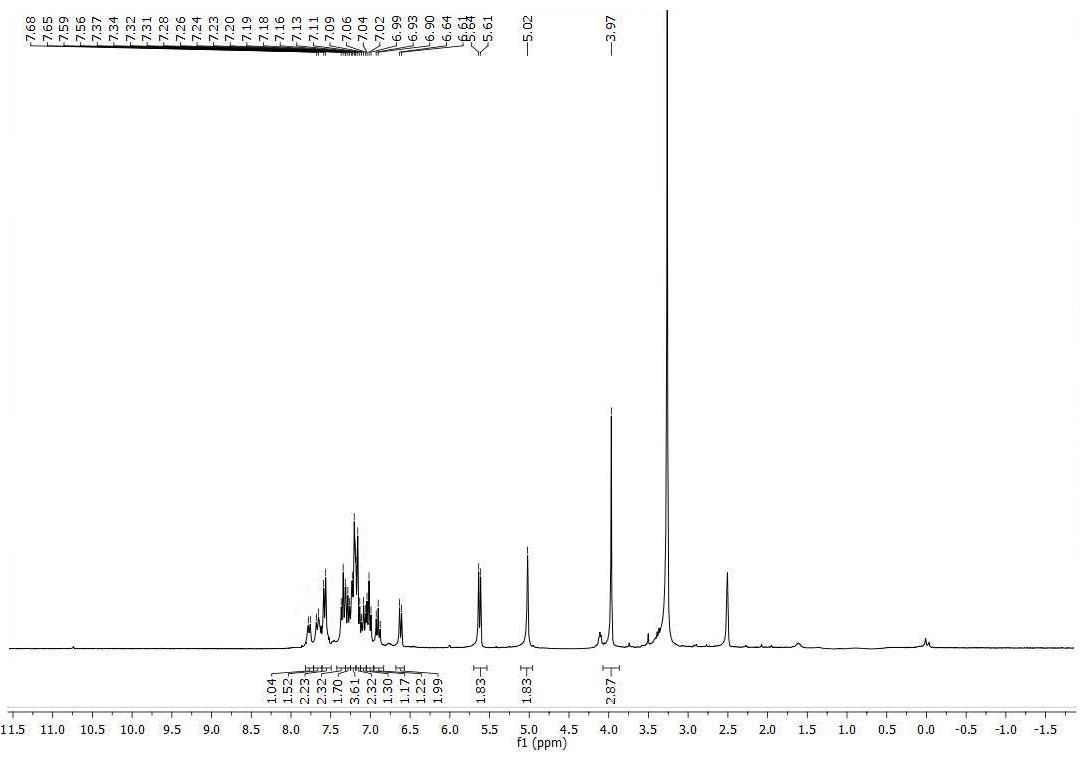
**

**Fig. 6.** ^1^H NMR spectrum of compound **4w** in DMSO-*d_6_.*

**
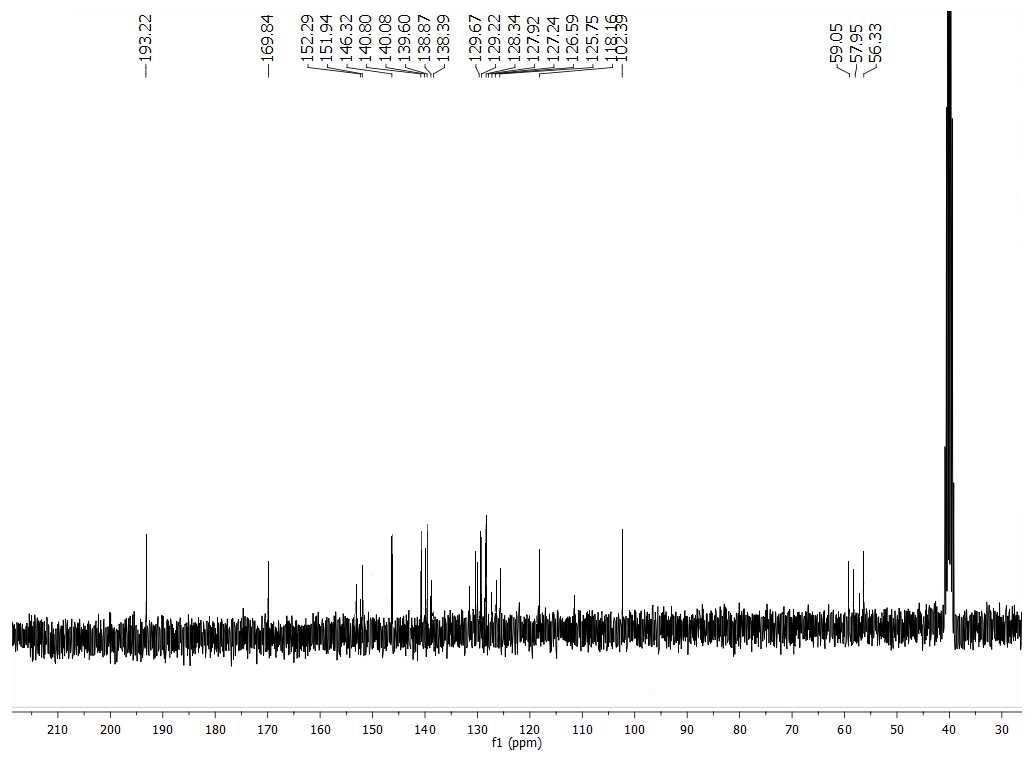
**

**Fig. 7.** ^13^C NMR spectrum of compound **4w** in DMSO-*d_6_.*

**Fig. 8.** FTIR spectrum of compound **4w** (KBr pellet).

**4)** 1′-(4-Bromobenzyl)-5-(4-methoxyphenyl)-5*H*-spiro[diindeno[1,2-*b*:2′,1′-*e*]pyridine-11,3′-indoline]-2′, 10,12-trione **(4x)**:

Yield: 80 %; Red powder; Mp > 300 °C; ^1^H NMR (300 MHz, DMSO-*d_6_*): δ (ppm) 3.97 (s, 3H, OCH_3_), 4.99 (s, 2H, CH_2_), 5.63 (d, *J =* 6.0 Hz, 2H, Ar-H), 6.64 (d, *J =* 6.0 Hz, 2H, Ar-H), 6.90 – 6.94 (m, 1H, Ar-H), 7.02 – 7.25 (m, 6H, Ar-H), 7.33-7.35 (m, 1H, Ar-H), 7.50 – 7.82 (m, 8H, Ar-H); ^13^C NMR (75 MHz, DMSO-*d_6_*): δ (ppm) 39.57, 40.30, 40.57, 40.98, 53.45, 56.33, 58.43, 102.95, 107.19, 109.72, 112.23, 113.58, 115.42, 116.86, 118.32, 121.15, 122.58, 123.61, 124.36, 125.72, 127.54, 129.31, 131.15, 137.12, 157.11, 160.26, 169.23, 190.21; FT IR (KBr pellet, cm^−1^): 3051, 1691, 1616, 1502, 1384, 1245, 1170; HRMS (ESI): *m/z* [M + Na]^+^ calcd. for C_40_H_25_BrN_2_O_4_: 699.0890, found: 699.0888.

**
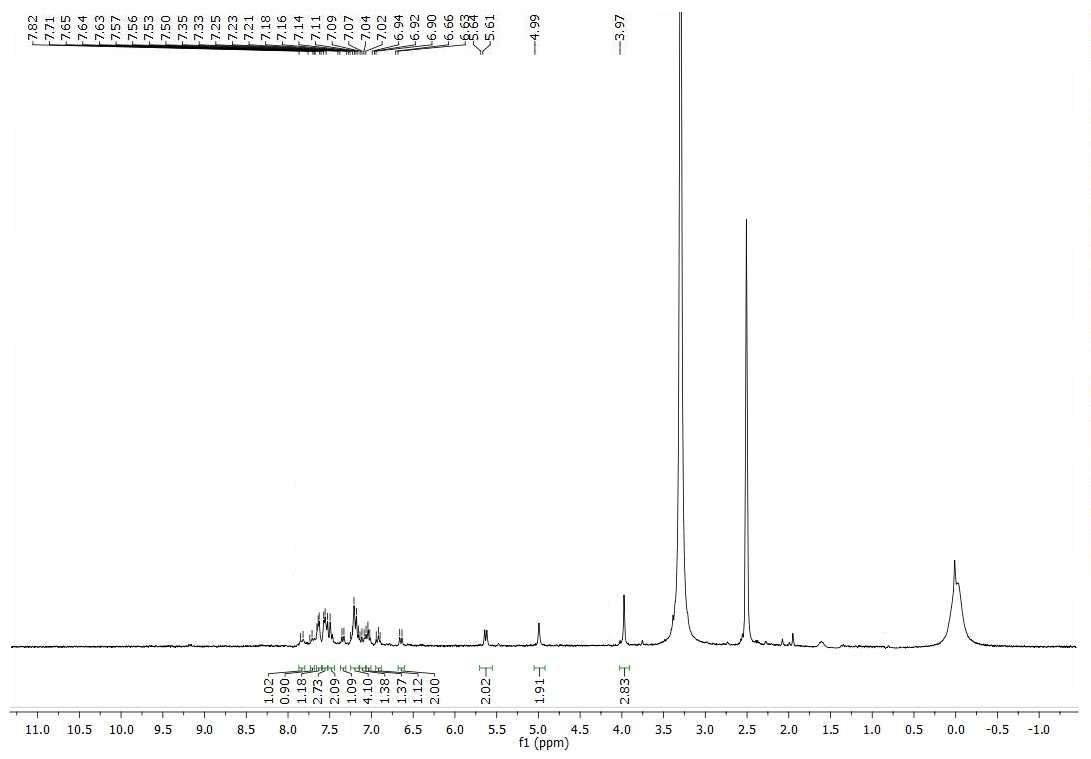
**

**Fig. 9.** ^1^H NMR spectrum of compound **4x** in DMSO-*d_6_.*

**
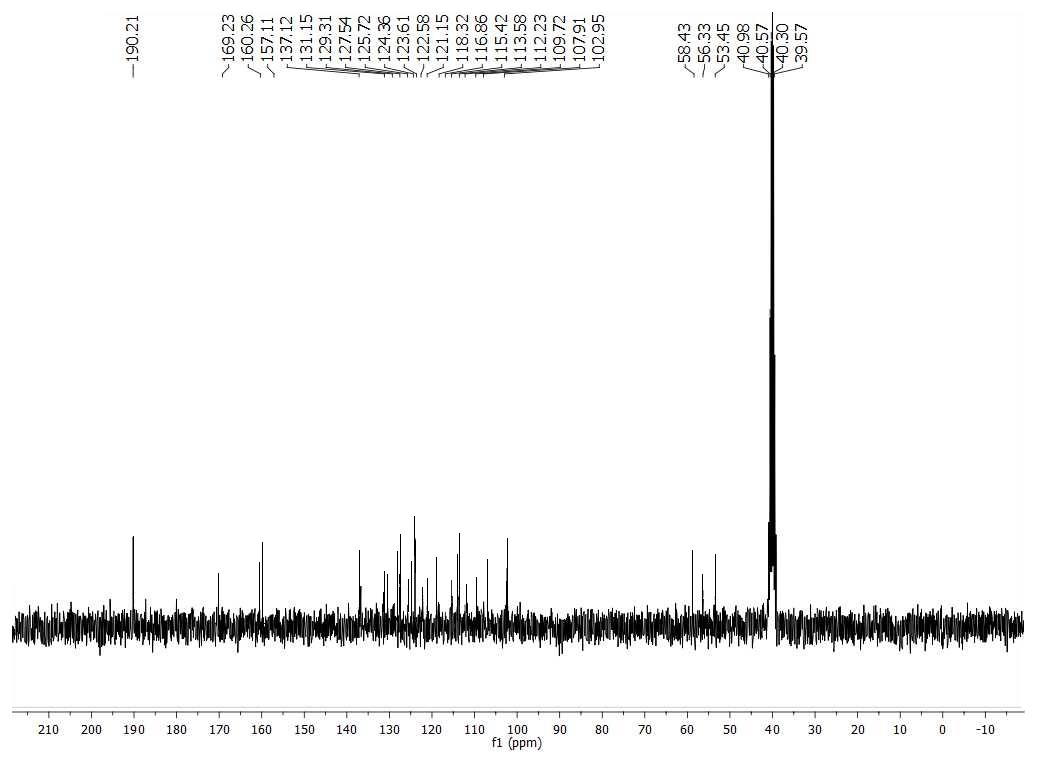
**

**Fig. 10.** ^13^C NMR spectrum of compound **4x** in DMSO-*d_6_.*

**Fig. 11.** FTIR spectrum of compound **4x** (KBr pellet).

**5)** 5-(3-Methoxyphenyl)-5*H*-spiro[diindeno[1,2-*b*:2′,1′-*e*]pyridine-11,3′-indoline]-2′,10,12-trione **(4y)**:

Yield: 94 %; Red powder; Mp > 300 °C; ^1^H NMR (300 MHz, DMSO-*d_6_*): δ (ppm) 3.87 (s, 3H, OCH_3_), 5.60 – 5.66 (m, 2H, Ar-H), 6.84 – 6.86 (m, 2H, Ar-H), 6.95 – 7.31 (m, 8H, Ar-H), 7.56 – 7.80 (m, 4H, Ar-H), 10.55 (s, 1H, NH); ^13^C NMR (75 MHz, DMSO-*d_6_*): δ (ppm) 38.13, 38.90, 39.71, 40.27, 40.54, 50.08, 55.10, 113.72, 116.56, 119.10, 121.30, 121.66, 123.84, 125.95, 127.44, 128.81, 130.68, 131.74, 132.86, 134.28, 136.43, 137.49, 148.05, 158.84, 162.15, 189.94; FT IR (KBr pellet, cm^−1^): 3367, 3076, 1695, 1620, 1386, 1242, 1147; HRMS (ESI): *m/z* [M + H]^+^ calcd. for C_33_H_21_N_2_O_4_: 509.1496, found: 509.1512.

**
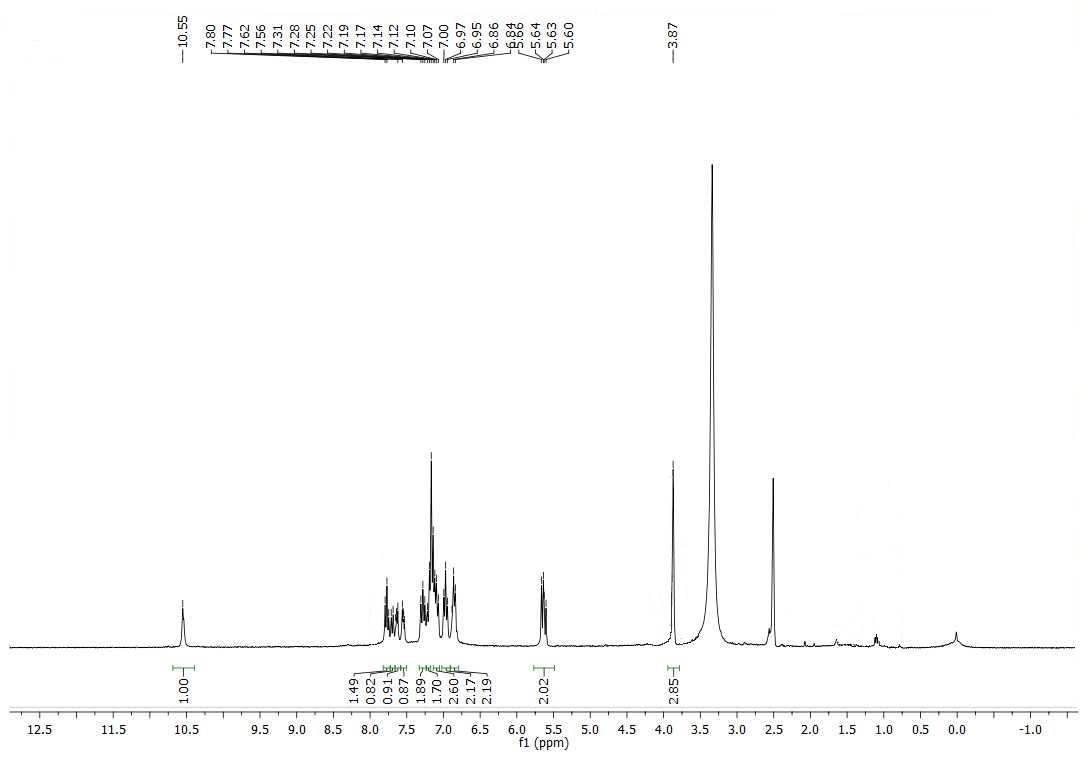
**

**Fig. 12.** ^13^C NMR spectrum of compound **4y** in DMSO-*d_6_.*

^
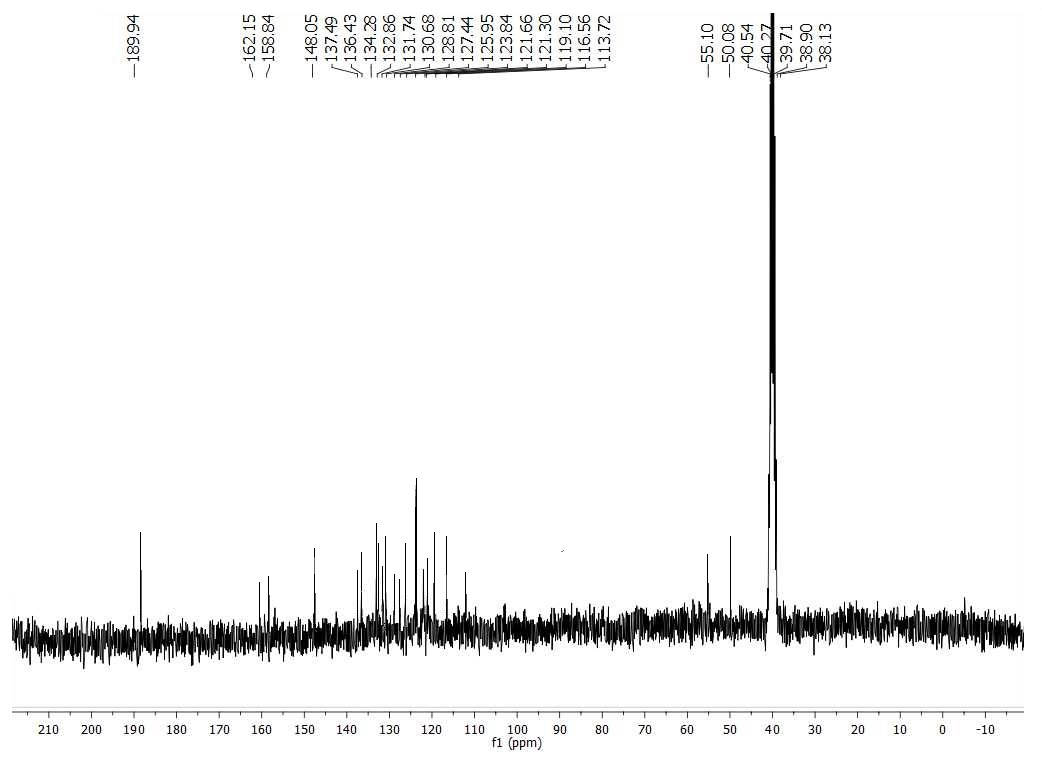
^

**Fig. 13.** ^13^C NMR spectrum of compound **4y** in DMSO-*d_6_.*

**Fig. 14.** FTIR spectrum of compound **4y** (KBr pellet).
